# One World Health: Neglected Tropical Diseases in a Flat World

**DOI:** 10.1371/journal.pntd.0000405

**Published:** 2009-04-28

**Authors:** Peter J. Hotez

**Affiliations:** 1 Sabin Vaccine Institute, Washington, D.C., United States of America; 2 Department of Microbiology, Immunology, and Tropical Medicine, George Washington University Medical Center, Washington, D.C., United States of America

In *The World Is Flat* and his other landmark books on globalization, journalist, columnist, and author Thomas Friedman eloquently articulates the prospect of a new world order and economy as a consequence of emerging new technologies, business practices, and world events [1]. Heading the list of “flatteners” that have leveled the new economic world are Netscape and the Web, common software platforms from Microsoft and elsewhere, search engines, open access and open sourcing, outsourcing, offshoring, supply chaining and insourcing, and mobile phones and other personal digital devices [1]. At the same time, globalization has also emerged as an important force in revealing the devastating health and economic impact of disease on human populations worldwide [2]. Through open access, *PLoS Neglected Tropical Diseases* is helping to increase awareness of the neglected tropical diseases (NTDs) as the most common infections of the “bottom billion,” i.e., the world's poorest people living on less than US$1 per day in developing countries [3]. By causing adverse effects on child development and learning, pregnancy outcome, and agricultural productivity, the NTDs are emerging as one of the most important group of infections that trap the poorest people living in sub-Saharan Africa, Asia, and the Americas in a cycle of poverty and destitution [2],[3]. The NTDs both occur in the setting of poverty and promote poverty [3].

New studies published in 2008 have also shown that in our newly flattened world the NTDs do not occur exclusively in developing countries [4]. Instead, wherever poverty is pervasive, even in otherwise wealthy countries such as the United States, some important parasitic and other infections are endemic, and they cause significant adverse effects on maternal and child health [4]. Shown in Table 1 is a comparison of some of these neglected diseases occurring in underrepresented minority African American and Hispanic populations in the US with populations living in Nigeria and Mexico, respectively. For example, the parasitic disease trichomoniasis is one of the most common infections adversely affecting reproductive health among African American women. Among this population in the US, the national prevalence of *Trichomonas vaginalis* infection was recently estimated at 13% [5], but it has been found to be as high as 29% among African American women attending a clinic for sexually transmitted diseases in Baltimore [6]. By comparison, in Ogun State, Nigeria, 38% of women attending a fertility clinic were found to be infected with *T. vaginalis* [7]. Similarly, the prevalence rate of toxocariasis, a parasitic worm infection linked to asthma and epilepsy, was recently estimated among African Americans nationally at 21% [8], compared with a prevalence of 30% in Plateau State, Nigeria [9].

**Table 1:** Comparison of NTD Prevalence and Incidence Rates between Selected Minority Populations in the United Sates and in Selected Developing Countries.

Neglected Tropical Disease	Underrepresented Minority US Population	Prevalence	Developing Country	Prevalence	Reference
Trichomoniasis	African Americans	13%–29%	Nigeria	38%	[4]–[7]
Toxocariasis	African Americans	21%	Nigeria	30%	[4],[8],[9]
HIV/AIDS	African Americans	2%	Nigeria	3%	[10],[11]
Cysticercosis	Hispanic Americans	2%	Mexico	44%	[4],[12],[13]
Dengue	Hispanic Americans	2%	Mexico	7%	[4],[14]
Invasive haemophilus disease	Native Americans	20.5 cases per 100,000 (age<5)	Mozambique	125 cases per 100,000 (age<5)	[20],[21]

Nearly 600,000 African Americans are living with HIV/AIDS and as many as 30,000 become infected each year [10]. In their August 2008 report, *Left Behind: Black America: A Neglected Priority in the Global AIDS Epidemic*, the Black AIDS Institute makes the poignant comment that if Black America was a country, its economy would be almost as large as that of South Africa, and that “a free-standing Black America would rank 16th in the world in the number of people living with HIV” [10]. They further state that in US inner cities and the American South the prevalence of HIV/AIDS approaches those reported in some of the most heavily affected countries in Africa [10]. Indeed, the prevalence of HIV/AIDS in Nigeria is only slightly greater than that in Black America [10],[11].

Cysticercosis, dengue, and Chagas disease are now endemic in the US among impoverished Hispanic populations, and in some cases the rates of these NTDs are comparable to those in poor regions of Latin America [4], [12]–[14]. Some of these cases in the US have emerged because of globalization and international migration [15],[16], but there is also strong evidence now for transmission of these parasitic diseases within US borders [4], [16]–[19]. The common denominator for neglected infections in the US such as trichomoniasis, toxocariasis, HIV/AIDS, cysticercosis, and Chagas disease, is poverty, not immigration [4]. Among Native Americans in the US, although invasive *Haemophilus influenzae* type b (Hib) infection is no longer common because of adequate vaccination coverage against this infection, invasive *H. influenzae* type a infection now occurs [20], and in some cases at rates that can be compared with Hib rates among unvaccinated populations in Africa [21].

The similarities between NTDs and HIV/AIDS in the US and in developing countries illustrate an interesting feature about Friedman's flat world. Just as aspiring nations such as India and China exploit new technologies that level their playing field with the US and Europe, the same playing field is (sadly) also being leveled on the other side. Even in wealthy countries such as the US, there are intense pockets of poverty where neglected infections can and do occur. As I pointed out previously, no one would argue that American poverty is nearly as pervasive or as intense as it is in sub-Saharan Africa or even in the poorest regions of Latin America or Asia [22], but it is nonetheless sufficiently severe among the estimated 36 million people living below the poverty line in the US for many poor Americans to be at risk for some important NTDs [4],[22]. It would also be worth exploring NTD rates in other parts of the developed world, such as those for leishmaniasis and other neglected infections in Europe [23],[24], intestinal helminth infections and ectoparasitic infections among aboriginal populations in Australia [25],[26], and anisakiasis, echinococcosis, strongyloidiasis, toxocariasis, and other parasitic and vector-borne NTDs in Japan [27]–[32]. An emerging global financial crisis in 2008 and 2009 is not likely to help this situation and could exacerbate even further infectious disease health disparities everywhere. The rates of non-communicable chronic diseases such as cancer and cardiovascular diseases would also be worth comparing between developed and developing countries.

Because NTDs are still largely forgotten diseases among forgotten people, we are at an early stage of having detailed information about their actual prevalence or incidence in both developing and developed countries. Accordingly, *PLoS Neglected Tropical Diseases* continues to solicit and review papers that measure disease burdens wherever poor people live [33]. In the meantime, awareness of the NTDs and their health and socioeconomic importance continues to increase among policymakers in the global public health community, while simultaneously funds are slowly increasing in order to provide support for access to essential medicines for NTDs among the world's poorest people. Given their importance in promoting poverty and the low-cost options now available for controlling or eliminating NTDs [2],[3], taking on these conditions may represent one of the most effective and cost-efficient solutions for addressing the Millennium Development Goals [2],[3],[34]. At the same time, it will be important to consider that in our flattened world it will be easy if not convenient to forget about NTDs in the US, Europe, Australia, and Japan. Our approach should include both addressing the social structures in which neglected infections flourish as well as research and development for new diagnostics, drugs, and vaccines. It will be a challenge to not leave the poor stranded among the wealthy, but in the world order outlined by Friedman, if the wealthiest countries wish to remain global competitors, individuals and societies in the lowest socioeconomic groups affected by neglected diseases (in any nation of the world, including the US) must be provided access to medicines and innovations for these conditions. This is also a fundamental issue in human rights [35] that we cannot ignore.
